# Spectacle-Glasses: When to Be Worn and How They Should Be Selected

**Published:** 1895-06

**Authors:** A. Bethune Patterson

**Affiliations:** Atlanta, Ga.; 14½ Whitehall St.


### SPECTACLE-GLASSES: WHEN TO BE WORN AND HOW THEY SHOULD BE SELECTED.

BY A. BETHUNE PATTERSON, M. D., ATLANTA, GA.

Spectacle-glasses are here considered as remedies, scientific remedies, based upon the mathematical condition of the eye, and the laws governing the refraction of light.

One who possesses some knowledge of the refraction of the eye, who knows the state of the standard or normal eye, (emmetropic) and the so-called errors or the departure from the normal, will readily comprehend what I propose to say on the subject of selecting and fitting glasses. But to more thoroughly comprehend the effects of glasses upon the refraction of the eye a knowledge of accommodation is essential. The accommodation is a physical condition, and is mathematically estimated, it has its expression in lens expansion and contraction. The lens is a transparent convex body, situated just in the rear of the iris or pupil, and is under the control of the ciliary muscle which completely surrounds it. Its range of movement varies with the age; thus at the age of ten, the range of expansion is fourteen dioptres, and is equal to convex lenses of that number; from the age of ten on there is a gradual loss in its dioptres of expansion. At twenty, there is only ten; at thirty, seven; at forty-five, three and a half; at fifty-five, one; while at sixty, one; though, practically, this one is not considered and is regarded as possessing none at all. Such an eye if emmetropic or normal would see objects distinctly at a distance, because it is adjusted for parallel rays—rays of light for practical purposes are considered parallel when coming from a luminous body, a candle, for instance, twenty feet away; these rays increase in divergence, as the candle is brought nearer to the eye, and the image of the flame, which at twenty feet was perfectly made upon the retina, gradually becomes more indistinct as it approaches the eye. So, for the eye to see the flame distinctly, or in fact any object, type for instance, when at nine inches, would require a glass of four or four and a half dioptres, that being the amount of lens expansion necessary to focus such divergent rays upon the retina. A child at the age of ten possessing a range of accommodation equivalent to fourteen dioptres would make use of four and a half to see the smallest letters distinctly at nine inches. So there would remain about ten dioptres unemployed, or at rest. It has been estimated that at least one-third or one-fourth of the accommodation should remain at rest, when an eye is being used for near work for any length of time, or fatigue and eye strain will follow. Thus a patient at forty-three has only about four dioptres of accommodation, which is sufficient to enable him to read at nine inches very fine type. It takes all he possesses to accomplish this, which could not be continued very long, on account of fatigue which would follow, but with the assistance of a plus one dioptre glass near work could be continued indefinitely without fatigue. So it is seen that one dioptre of his lens expansion has been put at rest, having been supplanted by the one dioptre glass. Now, let us suppose that the patient is not emmetropic, but far-sighted or hyperopic, say to the degree of one dioptre what would be the glass necessary for easy near work, reading, for instance? From the above it is seen that a one dioptre glass is necessary to cover his one dioptre of presbyopia, and with one dioptre of hyperopia he fails to read fine type at nine inches. The attempt gives the same degree of eye fatigue as our emmetropic patient, but with a plus two, one for his hyperopia, and one for his presbyopia, reads the finest type at nine inches. The hyperopia does not increase with advancing years, while the presbyopia gradually increases. At the age of forty-five, an emmetrope would require a plus two glass; at fifty, a plus three, at fifty-five a plus four; at sixty, plus five. Objects are only seen clearly when their shadows are accurately formed upon the yellow spot of the retina; such a shadow can only be formed by an accurate focusing of rays of light. So the indistinctness of objects is in proportion to the distance from the retina of these focal points. In hyperopia these points are in the rear of the retina, while in myopia or near-sightedness, the focal points are in front of the retina, and the indistinctness of objects at twenty feet away would be in proportion to the distance of the fo us in front of the retina; the point would be one millimeter in front of the retina in an eye of three dioptres of myopia, and would require a minus three dioptre glass, to throw back such focal points upon the retina, and a six dioptre glass would remove these points two millimeters. So in hypermetropia of three and six dioptres the foci would be respectively one and two millimeters behind the retina. In the latter or hyperopic condition accommodation is always necessary to see objects at a distance clearly, while in myopia the slightest action on the part of accommodation increases the myopia. Myopic eyes are not considered healthy eyes, and it is found best to excite their accommodation as little as possible, therefore much judgment is required in selecting glasses for such eyes, as the glass that would bring their accommodation into action would tend to increase the myopia, which would be undesirable. Astigmatism is a very troublesome condition, frequently taxing the skill of the most experienced refractionist. In regular astigmatism one-half of the eye focuses rays upon the retina; this is the emmetropic meridian, while the other half of the eye focuses either behind or in front of the retina, and is the hyperopic, or myopic meridian. In the former we have simple hypermetropic astigmatism, while in the latter, simple myopia astigmatism. Compound hypermetropic astigmatism is where both focal points are in the rear of the retina, and separated; in compound myopic astigmatism, the two focal points are at some distance from each other and both in front of the retina. Mixed astigmatism is where one meridian focuses rays behind the retina, and the other in front of retina. The remedy in this form of astigmatism would be a glass so ground as to draw forward the hyperopic meridian just far enough to rest upon the retina, and the other half of the glass ground concave to a degree necessary to throw rays back upon the retina, thus evening the focus of both meridians. In the simple hyperopic astigmatism, the glass for the correction is called a plus cylinder —only one-half of the glass is convex, while the other half is plane. This cylinder brings forward the hypermetropic rays on the retina. The above conditions of the eye when uncorrected are responsible for a great many troublesome symptoms, which harrass and make the lives of patients unhappy. A few of the most common will suffice here. First we have conjunctivitis, which is an inflammation of the mucus surface of the lids, and which is very common. A patient with this inflammation generally comes complaining of a rough or gritty sensation in eyes, a burning and smarting, eyes water, bright lights are distressing, and any near work increases these symptoms. Eyeballs ache and feel tired, lids feel heavy and there is a tendency to close them. Headaches are perhaps the most distressing symptoms met with, and it is said that glasses cure more headaches than the various drugs resorted to for the relief of pain. To relieve these conditions the errors of refraction must be corrected: the remedv for this correction is glasses. Before we can determine upon the correcting glass, it is necessary to set at rest the muscles that control the lens. This is done by instilling into the eye drops that possess a mydriatic effect, drugs that paralyze the muscles of accommodation, belladonna, scopolamine, and others. When the eye is thoroughly under the influence of such a remedy, the lens is at rest, and is as thin and flat as possible.

We proceed with the test, and find that the patient has simple hypermetropic astigmatism, say of one dioptre; the focus of the horizontal meridian is projected one-third of a millimeter behind the retina, while the focus of the vertical rests upon the retina, and we order a pluss cylinder of one dioptre, which only affectst he horizontal meridian, and evens the focus; both now resting upon the retina. Before we paralyze the patient's accommodation, the test would probably show a reversal of this, the accommodation acting to draw forward the hypermetropic meridian would also draw forward the emmetropic meridian one dioptre. So our test would show simple myopic astigmatism, though sometimes it appears that the muscles of accommodation act irregularly. In simple hypermetropia the accommodation acting would conceal the degree, so it would be impossible to ascertain the error without the use of a mydriatic. Whether or not the case is one which necessitates wearing glasses constantly or only for near work and if the glass which corrects the eye for distant vision will be satisfactory for doing near work, can only be determined upon after considering age of patient and amount of accommodation possessed. The duty of the occulist does not rest with the writing of prescription for glass decided upon; he must see that the glasses are put in frames which fit the patient, and hold the glasses in proper position before the eyes. This we deem as important as finding the correcting glass. The spectacle frame should be so adjusted that the patient looks through the center of his glass. It is important that the two centers correspond, because the rays of light that fall upon the eye are changed in their direction, except the center rays, which pass straight through the eye without being deflected. so it is with the rays passing through a lens. All are changed except the center rays. The position then of the glass would not be the same in the respective conditions of near and distant vision. When the glass is adjusted for distance, the patient looks through the center when looking straight ahead, while in reading with this glass, he would look some distance below the center. Such a glass in reading would act as a prism, and unless very weak, would strain the eye muscles and give trouble. When the frames are adjusted for near work the patient, when observing some object at a distance, would look several millimeters above the center. The following is from an article entitled "Glaucoma," which I wrote, and had published in the *Southern Medical Record* of November, 1893 and which bears upon this point: "Recognizing the disease, glaucoma to be one of advanced life, we naturally look to the presbyopic conditions as playing a part in the etiological role. We are all familiar with the pain in eye and head and the congestion of the conjunctival vessels so common in eye strain due to astigmatism, hyperopia, and presbyopia, and when accompanied by a run-down condition of health, a gouty and rheumatic dyscrasia seems to be quite enough to bring about the degenerative condition of the eye tunics. Every effort should be made to eliminate eye strain, astigmatism, hyperopia, presbyopia, and the degree should be estimated and accurately corrected with properly adjusted glasses. I wish to accentuate the phrase "properly adjusted." Care should be exercised in selecting frames, as the correct position of the glass must depend upon a correctly adjusted frame. Patients should look through the center of their glass; if above or below this point, or out of the corners of the glass, eye strain will surely result. Glasses should never be selected by patients, and under no circumstances, should this important piece of scientific work be entrusted to the optician." The fitting of glasses does not come under the domain of the optician and spectacle vender, any more so than the remedies on the shelves of the apothecary are his to prescribe for diseased conditions. The fact is the laws of the country forbid such prescribing, then why should not the laws be made to apply as well to the optician. Such legislation would prevent innocent people from being injured and imposed upon. The advertisements of opticians to "scientifically test eyes and fit to glasses," while very seductive, is fallacious. Glasses have a still broader range of usefulness in asthenopia, or eye strain, which are not due to refraction errors, but to abnormal or weakened conditions of the muscles which control the movements of the eyeball; these muscles are the internal and external recti, the superior and inferior recti, and the obliques. Many cases of strabismus, or cross-eyes, if taken in time are cured by spectacles, which correct the errors of refraction.

The advantages spectacles possess over eye-glasses are generally overlooked except by the occulist, who rarely ever orders eye-glasses. The principal objections to the latter are found in their unsteadiness, and the difficulty in getting them properly centered. They have their field of usefulness, however, and can be ordered when glasses are only to be used for a short time, or when the occupation requires them to be put on and off at short intervals. In adjusting glasses for distance, it should be remembered that the glass should not be tilted, but the plane of glass and plane of face should be parallel, while in glasses for near work, reading and writing, the two planes should not correspond while the head is erect and patient looking straight ahead. The glass is now tilted at an angle of five to ten degrees. At this angle the two planes, that is, of the face and glass, will become parallel when the position of reading and writing is assumed. This is important and should never be overlooked.

*14½ Whitehall St.*

